# A Cluster Randomized Controlled Trial Evaluating the Impact of Tailored Feedback on the Purchase of Healthier Foods from Primary School Online Canteens

**DOI:** 10.3390/nu13072405

**Published:** 2021-07-14

**Authors:** Fiona Stacey, Tessa Delaney, Kylie Ball, Rachel Zoetemeyer, Christophe Lecathelinais, Luke Wolfenden, Kirsty Seward, Rebecca Wyse

**Affiliations:** 1 Hunter New England Population Health, Locked Bag 10, Wallsend, NSW 2287, Australia; Tessa.Delaney@health.nsw.gov.au (T.D.); rachel.zoetemeyer@newcastle.edu.au (R.Z.); Christophe.Lecathelinais@health.nsw.gov.au (C.L.); Luke.Wolfenden@health.nsw.gov.au (L.W.); kirsty.seward@newcastle.edu.au (K.S.); Rebecca.Wyse@health.nsw.gov.au (R.W.); 2 School of Medicine and Public Health, University of Newcastle, Callaghan, NSW 2308, Australia; 3 Priority Research Centre for Health Behaviour, University of Newcastle, Callaghan, NSW 2308, Australia; 4 Hunter Medical Research Institute, New Lambton Heights, NSW 2305, Australia; 5 Institute for Physical Activity and Nutrition, Deakin University, Burwood, VIC 3125, Australia; kylie.ball@deakin.edu.au

**Keywords:** randomized controlled trial, nutrition, primary school, tailored feedback, online, digital health intervention

## Abstract

Few online food ordering systems provide tailored dietary feedback to consumers, despite suggested benefits. The study aim was to determine the effect of providing tailored feedback on the healthiness of students’ lunch orders from a school canteen online ordering system. A cluster randomized controlled trial with ten government primary schools in New South Wales, Australia was conducted. Consenting schools that used an online canteen provider (‘Flexischools’) were randomized to either: a graph and prompt showing the proportion of ‘everyday’ foods selected or a standard online ordering system. Students with an online lunch order during baseline data collection were included (*n* = 2200 students; *n* = 7604 orders). Primary outcomes were the proportion of foods classified as ‘everyday’ or ‘caution’. Secondary outcomes included: mean energy, saturated fat, sugar, and sodium content. There was no difference over time between groups on the proportion of ‘everyday’ (OR 0.99; *p* = 0.88) or ‘caution’ items purchased (OR 1.17; *p* = 0.45). There was a significant difference between groups for average energy content (mean difference 51 kJ; *p*−0.02), with both groups decreasing. There was no difference in the saturated fat, sugar, or sodium content. Tailored feedback did not impact the proportion of ‘everyday’ or ‘caution’ foods or the nutritional quality of online canteen orders. Future research should explore whether additional strategies and specific feedback formats can promote healthy purchasing decisions.

## 1. Introduction

Globally, poor diet is a leading and modifiable risk factor for non-communicable diseases such as cardiovascular disease, some cancers, type 2 diabetes and obesity, with 7.95 million deaths and approximately 7% of DALYs attributable to dietary risk factors in 2019 [1]. Poor dietary patterns are often established during childhood and continue into adulthood [2], and are associated with increased risk of chronic disease [3]. Poor diet is prevalent in children, with few children internationally meeting recommended dietary intake guidelines [2,4,5]. For example, foods high in added sugars and fats contribute up to 40% of children’s total energy intake in the US and Australia [6,7]. Given the prevalence of poor nutrition in children, and the impact of nutrition on health outcomes, there is a need to explore effective and feasible approaches to improve public health nutrition.

Tailoring personalizes the content and format of information an individual receives, based on characteristics unique to that person [8]. Tailoring influences the degree to which people attend to information, reflect on it, find it relevant and salient, and their intention to act upon that information [9]. The impact of tailored nutrition information may be enhanced through easy to understand feedback that facilitates comprehension. Using simple visual feedback, such as pie graphs [10] and color-coded labels [11], can assist in comprehending information and can improve the nutritional quality of food purchases [10,11]. Studies of both children and adults have indicated that a single pictorial summary of nutrition quality (such as a health star rating) is the preferred front-of-pack nutrition label on products [12,13], compared to percentage daily intake guides, or multiple traffic lights. 

Evidence from systematic reviews indicates that tailored nutrition interventions in adults have a greater effect on improving nutrition-related outcomes than non-tailored approaches [14,15]. For example, meta-analyses on the long term effect of tailored compared to generic nutrition education found that tailored approaches were associated with an increase in daily fruit and vegetable consumption and a decrease in energy consumed from total fat [15]. Tailored messages and feedback have also been identified as characteristics that contribute to effective interventions in reviews of digital or online interventions targeting children and adolescents [16] and adults [17]. A recent meta-analysis of 19 e-health interventions on fruit and vegetable effectiveness demonstrated a greater effect among tailored interventions [18]. This evidence suggests that tailored interventions have the potential to play an important role in effective on- and offline nutrition interventions. 

One promising opportunity to deliver tailored nutrition feedback to improve public health nutrition is via online food ordering platforms. Such platforms allow users to view, select and order food and drink items online, and are now common ways of accessing groceries, fast food, meals, snacks, and beverages, and they continue to increase worldwide both in popularity and revenue by 6% annually [19]. Online food ordering platforms offer an appealing real-world opportunity to apply strategies to promote behavioral nutrition that can reach millions of consumers at a relatively low cost and at high fidelity, at the key decision making point, could be tailored to the individual, and could be modified in real time [20,21]. Furthermore, the flexibility of digital presentation means that they can be dynamically displayed to encourage people to attend to feedback. However, evidence from these routinely used ordering systems is scarce. A US study compared the lunch orders of 5th and 6th grade students who pre-ordered their lunch via computer [22] with students who pre-ordered and received tailored feedback and a visual display of their order compared to the food group recommendations [22]. Students who received the tailored feedback and visual display significantly increased their purchase of fruits, vegetables, and low-fat milk compared to controls [22]. While this study indicates the potential of this strategy to improve the nutritional quality of foods ordered online, it was conducted in a single school over a 2-week period, within a purpose-built online ordering system, rather than being tested within an existing ordering system with an established user-base, and additional research is warranted.

Online canteens are a form of online ordering platform used for children to access food at school. They have the potential to deliver nutrition interventions to this priority population [23]. For example, almost 1400 Australian schools and over 400,000 users access the leading online canteen lunch ordering system in schools [24,25]. A recent cluster randomized controlled trial within this setting established that a multi-strategy intervention which included individualized feedback in the form of a pie graph was effective in increasing the purchase of healthier (‘everyday’) items and reducing the energy and saturated fat content of student online lunches [26]. However, the study was unable to determine the effectiveness of the individual strategies, including the provision of tailored pie graph feedback. 

Given the effectiveness of tailored feedback in other settings, and the potential reach of online canteens [25] and online food ordering platforms more generally [19], there is a need to test the effectiveness of tailored feedback in a real-world online ordering system. We aimed to evaluate the effects of embedding tailored feedback and a graph within a real-world online canteen to improve the healthiness of the lunch order purchases for primary school students. 

## 2. Materials and Methods

### 2.1. Design

The study was a parallel group cluster randomized controlled trial. Schools (clusters) using an online canteen with embedded online labels (colored labels next to each menu item to indicate whether the item was categorized as an ‘everyday’, ‘occasional’, or ‘caution’ food according to NSW Healthy School Canteen Strategy [27]) were randomly assigned to receive either a 4-week tailored feedback intervention, or control (no change to the existing online ordering system). As this intervention was designed specifically for the users of the online ordering system (parents, carers, and students), rather than the canteen manager, a minor modification was made to the labelling of the classification system so that it was more appropriate for consumers, rather than suppliers (canteen managers). Specifically, items categorized as ‘should not be sold’ under the NSW Healthy School Canteen Strategy were re-labelled as ‘caution’.

### 2.2. Participants and Setting

Government primary schools within NSW, Australia, with an online canteen and that had previously been recruited to participate in a randomized controlled trial of nudge strategies to encourage healthier purchasing from online school canteen ordering systems were approached to participate. The study was approved by the NSW State Education Research Applications Process (SERAP) 2018065 and the University of Newcastle Human Research Ethics Committee (H-2017-0402), and was retrospectively registered with the Australian and New Zealand Clinical Trials Register (ACTRN12619001150134). 

School recruitment took place from October to December 2018. Schools were eligible to participate if they used an existing online canteen service provided by ‘Flexischools’. Flexischools is the largest provider of online canteen services in Australia, servicing over 1200 schools, and processing over 13 million lunch orders in 2018 [24]. Users of the online canteen ordering system could be students or parents who place an order on behalf of their child. Users were included in the study if they placed an online lunch order in the 4-week baseline period (5th–30th August 2019).

#### Exclusion Criteria 

Schools: School canteens that were operated by a private operator (i.e., were ‘externally licensed’) were excluded given these operators often service multiple schools, thus would increase the potential for contamination between schools within the trial. Schools who had, within the previous three years, participated in trials by the research team involving fieldwork or site visits were excluded as required by the ethics committee. 

Users: Lunch orders that were pre-ordered prior to the commencement of the intervention period (e.g., recurring student orders) were excluded, as the user would not have been exposed to the feedback while placing the order. All orders that weren’t placed for students in kindergarten to year six (i.e., orders for teachers, school administrative staff and guests) were also excluded. Orders with an implausible number of items that could be consumed by one person (e.g., 15 ice blocks) were also excluded based on the review of orders and consensus from dietitians with extensive school canteen experience. Orders that were placed from a desktop device were also excluded as the tailored feedback was only visible on orders placed via a mobile device. 

### 2.3. Context

Government schools in NSW are subject to the NSW Healthy School Canteen Strategy, which categorizes food and drinks as ‘everyday’, ‘occasional’ or ‘caution’ and stipulates that the canteen menu be comprised of at least 75% ‘everyday’ items, no more than 25% ‘occasional’ items and 0% ‘caution’ items [27]. The NSW Healthy School Canteen Strategy [27] was introduced in 2017 with the requirement that Government school menus be compliant by December 2019. All participating schools had been recruited to an earlier trial testing an audit and feedback report of the menu provided to the school canteen manager and a color-coded menu labelling strategy (ACTRN12620001284954) (results reported separately) and were re-randomized prior to the current trial. All participating schools had previously received: (i) a feedback report comparing their canteen menu to the Strategy (delivered between 18 February 2019 to 3 April 2019; approximately 4–5.5 months prior to baseline) and (ii) menu labelling (implemented between 28 March 2019 and 5 June 2019; approximately 2–4.5 months prior to baseline) that consisted of adding a colored symbol denoting either ‘everyday’, ‘occasional’ or ‘caution’ classification next to each menu item and a key added explaining each of the symbols. Following delivery of these strategies for the previous trial, there was an eight week wash-in period where all participating schools received no further intervention, and the intervention support provided up to the start of the wash-in period was equivalent for all schools. The current trial was undertaken in weeks 3–10 of Term 3, 2019 (5 August 2019 to 27 September 2019). 

### 2.4. Intervention

**Figure 1:**
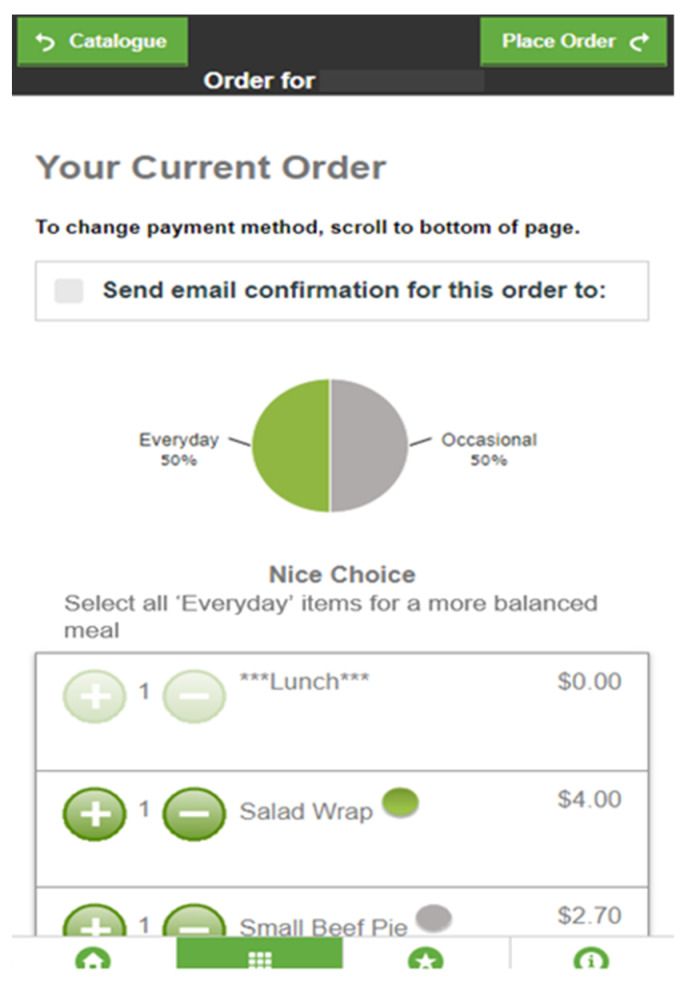
Feedback screen showing pie graph feedback.

All users of the online canteen system at intervention schools received a tailored feedback message and a graph based on the proportion of everyday items in the lunch order based on the Australian Guide to Healthy Eating [28] and the NSW Healthy School Canteen Strategy [27] (Figure 1). Using simple and visual feedback, such as pie graphs [10] and color-coded labels [11] can assist in comprehending information and can improve the nutritional quality of food purchases [10,11]. The intervention strategy was developed based on previous reviews of computer-generated personalized nutrition education [29], and is illustrated in Figure 1. 

After selecting menu items in the online canteen but prior to finalizing the lunch order, the user was taken to a feedback screen that displayed the pie graph showing the proportion of ‘everyday’, ‘occasional’, and ‘caution’ items contained within their lunch order. In addition to the graph, participants also received a brief message tailored to the proportion of ‘everyday’ items in their order:

0% ‘Everyday’ items: “Try adding some ‘Everyday’ items for a more balanced meal”
1–49% ‘Everyday’ items: “Good start—add some more ‘Everyday’ items for a more balanced meal”
50–99% ‘Everyday’ items: “Nice choice—select all ‘Everyday’ items for a more balanced meal”
100% ‘Everyday’ items: “100% ‘Everyday’ items—Excellent Choice!”

The feedback screen also included links to additional information about the NSW Healthy School Canteen Strategy. Users were able to amend their order at this point and the graph automatically was updated to reflect the new order. The intervention was programmed into the online system by Flexischools and they switched this feature on centrally following randomization (and at the request of the researchers). 

### 2.5. Randomization and Blinding

An independent statistician (CL) used a random number function in Microsoft Excel to randomize schools to either an intervention or control group in a 1:1 ratio. Schools were re-randomized following a previous trial, and were stratified based on previous allocation. The sample had previously been stratified based on the Socio-Economic Indexes for Areas (SEIFA) [30], with postcode used to classify schools as either ‘higher’ or ‘lower’ socioeconomic index based on the NSW median. Participating schools and users were not blinded to the intervention. The data analyst was not blinded to study group.

### 2.6. Primary Outcomes

The two co-primary trial outcomes were the:

(i). proportion of all lunch order items purchased that were classified as ‘everyday’, based on nutritional assessment of purchasing data automatically collected by the online canteen.
(ii). proportion of all lunch order items purchased that were classified as ‘caution’, based on nutritional assessment of purchasing data automatically collected by the online canteen.

‘Occasional’ items were not included as an outcome as changes to the proportion of items in this category can represent clear positive and negative outcomes (depending on whether the change is from the ‘caution’ or ‘everyday’ category).

To assess the primary outcomes, a dietitian classified all menu items at all schools using the NSW Healthy School Canteen Strategy. All purchases were recorded automatically by the online canteen at baseline (weeks 3–6, term 3 2019) and follow-up (weeks 7–10, term 3 2019). The statistician then applied these classifications to the purchasing data to establish the proportion of all items that were classified in each category. 

### 2.7. Secondary Outcomes

The secondary trial outcomes were the: 

Nutritional content: Mean energy (kJ), saturated fat (g), sugar (g), and sodium (mg) content per student online lunch order, based on nutritional assessment of purchasing data that was automatically collected by the online canteen. For pre-packaged foods, this was based on a dietitian’s classification with reference to the following sources (in order of use): 1. a database of over 2000 commonly stocked canteen products developed by canteen researchers over the past 5 years; 2. The FoodFinder database [31]; 3. The FoodSwitch website [32]; 4. An online search for the nutrient information panel. For canteen-made food, the recipe was obtained from the canteen manager and analyzed using the FoodWorks nutrition analysis software (Version 9) [33]. The statistician then matched the nutritional content to the automatically collected purchase data. 

*Canteen revenue:* Purchasing data automatically collected by the online canteen was used to calculate mean weekly online lunch revenue per school (adverse outcome).

### 2.8. School Characteristics

Data regarding school characteristics were obtained from a publicly available national school dataset (www.myschool.edu.au, accessed on 26 November 2018) [34] with the following information extracted: number of student enrolments, proportion of Aboriginal and Torres Strait Islander enrolments, and postcode. 

### 2.9. Sample Size Calculation

Recruitment of 10 schools and 200 students per school would allow detection of a 19.5% significant difference in the purchase of ‘everyday’ items, assuming an Intraclass Correlation Coefficient (ICC) of 0.05, with 80% power, and 0.05 significance level. An effect of this size has been previously found in a study of 10 government schools where 5 were receiving a multi-component intervention to improve the healthiness of student lunch orders [35]. Similarly, the study was powered to detect a 13.5% significant decrease in the purchase of ‘caution’ items. 

### 2.10. Analysis

All data were analyzed in SAS version 9.3 (SAS Institute Inc., Cary, NC, USA). Descriptive statistics were used to describe the school (enrolments, proportion of Aboriginal or Torres Strait Islander students, socio-economic status), canteen (type of canteen operation, days of operation, mean number of lunch orders) and student characteristics (grade and frequency of canteen use). 

We used an intention-to-treat approach whereby all student orders and schools were analyzed based on the groups to which they were originally allocated, and included data from all students that had baseline purchasing data. Each primary outcome was assessed using a separate logistic mixed model (i.e., items that are ‘everyday’ versus items that are not ‘everyday’) comparing intervention and control groups over time (between baseline and follow-up) by including a group by time interaction fixed effect. Each secondary trial outcome was assessed using a separate linear mixed model. The energy, saturated fat, sugar, and sodium content of all online lunch orders placed by students was compared between intervention and control groups over time by including a group by time interaction fixed effect. All models included a random intercept for school (to account for potential school level clustering), a nested random intercept and random time effect for students (to account for student level clustering and repeat measurements), and fixed effects for SEIFA. 

## 3. Results

**Figure 2:**
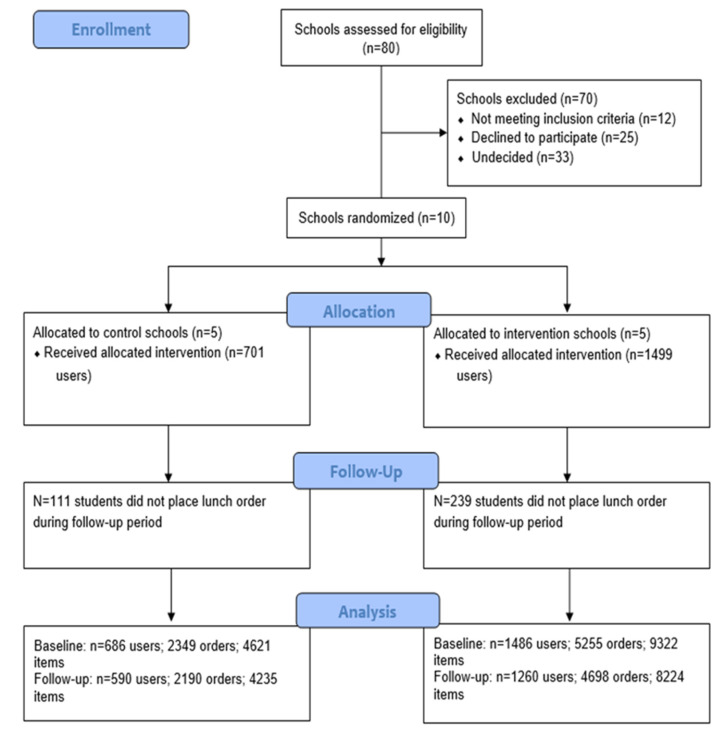
Participant Flow (CONSORT).

The CONSORT diagram shows the progress of schools through the trial (See Figure 2). Of the 80 schools assessed for eligibility, 12 were ineligible (two had participated in fieldwork or site visits for other research trials within the last 3 years and 10 were externally licensed). After nine weeks of recruitment, 10 of the eligible 68 schools had consented (15%), 25 had refused (37%) and 33 (49%) were undecided. Five schools were randomized to the intervention group and five were randomized to the control group. No schools dropped out over the intervention period.

The characteristics of the participating schools and users of the online canteen system at baseline are described in Table 1. Intervention schools had a higher number of mean enrolments per school (mean = 574) compared to the control group (mean = 460). The five intervention schools placed 5255 orders, consisting of 9322 items, for 1499 students during the baseline period, with an average weekly order frequency of 0.88 (SD 0.72) orders per student. The five control schools placed 2349 orders, consisting of 4621 items, for 701 students during the baseline period, with an average weekly order frequency of 0.84 (SD 0.67) orders per student. No schools (clusters) dropped out of the study. 

*Exclusions:* The following orders were excluded: recurring orders (*n* = 357); implausibly large orders (*n* = 1); and orders that were placed on a device other than mobile phone (*n* = 981). 

### 3.1. Primary Outcomes

At baseline, analysis of the purchasing data showed that intervention group online lunch orders had 56.03% ‘everyday’ and 8.81% ‘caution’ items. At baseline, control group lunch orders had 55.90% ‘everyday’ and 1.42% ‘caution’. At follow-up, the logistic mixed model showed no difference over time between the intervention and control group in the proportion of ‘everyday’ (OR 0.99 [95% CI 0.87, 1.13]; *p* = 0.88) or ‘caution’ items purchased (OR 1.17 [95% CI 0.73, 1.88]; *p* = 0.45) (See Table 2). This equated to non-significant differences in the anticipated direction of 1.5% and −0.3% between the groups for ‘everyday’ and ‘caution’ items, respectively.

### 3.2. Secondary Outcomes

There was a small but significant difference between intervention and control groups over time in the average energy content of lunch orders (mean difference 51 kJ [95% CI 11, 90]; *p* = 0.02). The energy content of orders in both the intervention and control groups decreased between baseline and follow-up. However, contrary to expectations, the decrease in the control group orders (−75 kJ) was greater than the decrease in the intervention group (−20 kJ). There was no difference in the nutritional content of lunch orders between the intervention and control group over time for saturated fat (mean difference 0.1 g [95% CI −0.1, 0.3]; *p* = 0.22), sugar (mean difference 0.1 g [95% CI −0.7, 1.0]; *p* = 0.75), or sodium (mean difference 12 mg [95% CI −9, 33]; *p* = 0.22). 

### 3.3. Adverse Outcome

There was no difference in the average weekly revenue from online lunch purchases between intervention and control schools over time (mean difference AUD 18.15 [95% CI -AUD 89.11, AUD 125.41]; *p* = 0.74.

## 4. Discussion

In this sample of users of an online canteen in primary schools, the pie graph and tailored feedback did not have an impact on the proportion of ‘everyday’ or ‘caution’ items purchased by users. These proportions remained similar between baseline and follow-up for both groups. Similarly, there was no difference between the intervention and control groups over time in the saturated fat, sugar, or sodium content of lunch orders. However, contrary to expectations, there was a small but significant between-group difference over time in the energy content of lunch orders, with the decrease in control orders exceeding the decrease in intervention orders (mean difference 51 kJ [95% CI 11, 90] *p* = 0.02). This difference in isolation may not have a substantial public health impact, but could represent an important contribution to a range of reductions in the daily energy intake of children arising from a comprehensive suite of public health interventions. As such, in this sample, the pie graph and tailored intervention did not improve the nutritional quality of student online lunch orders. 

These results are in contrast to a similar trial of a behavioral intervention delivered via an online school lunch ordering system. Miller et al., 2016 [22], reported that students who received a tailored feedback message comparing their menu selection against five food groups and a graphical representation of their plate increased their selections of fruits, vegetables, and low-fat milk from an online lunch ordering system [22]. A key difference between the two trials is that the Miller et al. (2016) intervention provided specific feedback on clearly identifiable food groups (e.g., fruit) [22], whereas the current intervention provided very broad, non-specific feedback (e.g., ‘everyday’ foods). It may be that tailored feedback related to a specific food group, such as fruits and vegetables, is easier to action and has a greater impact on food selection and purchase. 

A further possible explanation of the findings could be that the content of the tailored feedback was not detailed enough to prompt changes to the order. In a trial comparing kilojoule labelling and kilojoule plus health star rating labelling on fast food menus, there was no difference on the mean kilojoule count of meals [36]. However, those adults who viewed both the heath star rating and the kilojoule labels selected healthier meals overall (based on the Nutrient Profiling Score) [36]. This could also indicate that more comprehensive feedback is required. 

In our current trial, despite small reductions in the energy content of lunch purchases, there was no change to the proportion of ‘everyday’ or ‘caution’ items purchased. It is possible that parents made swaps for less energy-dense purchases, but that these swaps occurred within the same category. There is large variability in energy content in the classification categories within the NSW Healthy School Canteen Strategy [27]. For example, the ‘everyday’ category could include a 1096 kJ lasagna as well as a 329 kJ apple. The evaluation of this intervention is based on the final items purchased. We did not have data about items that were selected but then swapped-out prior to finalizing the order. More detailed analytic data tracking of the addition of items to the online basket would provide useful insight into participant responses to the intervention and is recommended for future research.

At baseline, the majority of purchases in both groups were already ‘everyday’ foods. Given this is the healthiest category within the classification system, it was not possible for users to swap to a healthier category. Despite this, future studies should consider more tailored strategies to encourage users to switch from ‘occasional’ to ‘everyday’ foods. Furthermore, prior to this study commencing, all participating schools had their menus labelled. This initial strategy may have informed and prompted change within users, leaving little room for subsequent improvement. 

The content of the prompts should also be considered in discussion of the study findings. There were four different prompts. The prompts were positive, i.e., when a combination of healthy and less healthy foods were selected (i.e., 1–99% ‘everyday’ items), praise was always given for the ‘everyday’ foods that were selected, before suggesting that users select more ‘everyday’ foods; e.g., “Good start—add some more ‘Everyday’ items for a more balanced meal” and “Nice choice—select all ‘Everyday’ items for a more balanced meal”. It may be that these prompts were not specific or direct enough to prompt action, and that the positive reinforcement from the first part of the prompt overwhelmed the subsequent call to action. As such, it is recommended that future research test more direct prompts. 

### Limitations and Strengths

The findings of the study should be considered in the context of its limitations and strengths. Trial limitations included a small sample of ten government schools with five schools in each study group. Despite randomization, there appear to be some differences between intervention and control schools. Intervention schools were larger (mean enrolments 574 compared to 460 in control schools) and therefore had a larger number of canteen orders, although the average number of orders per student per week was similar between the two groups (mean 0.9 orders per student per week in intervention schools compared to 0.8 orders per student per week in control schools). Future larger trials may wish to consider stratifying based on school size to ensure a similar numbers of students and orders between groups. There also appear to be differences in the purchase of ‘caution’ items at baseline between the intervention and control schools, with the intervention group reporting a higher proportion of ‘caution’ items purchased (8.8% compared to 1.4% in the control group). However, during the time the study was undertaken (2019) there was a mandatory canteen policy that government schools were required to remove all ‘caution’ items from their canteen. All government schools were required to work toward achieving compliance with this policy and were offered support via their Local Health District to implement this policy. The dietitian who analyzed the data may have become unblinded to group allocation as they were also involved in undertaking fidelity checks in intervention schools. Finally, these schools were part of an earlier trial testing the effect of nutrition labelling. However, all schools had previously received all of the same strategies and following this trial, there was a washout period of eight weeks before the commencement of the current study.

Strengths of the study include the cluster randomized controlled design. The online intervention strategies were applied directly to all orders through the online canteen and centrally switched on by Flexischools, resulting in high intervention fidelity. The purchasing data that formed the primary and secondary outcomes was recorded automatically by the online canteen, and was not subject to any self-report or recall biases. The trial collected a large amount of data with 7604 lunch orders for 2200 students placed during the baseline period. Furthermore, the trial was undertaken using a real-world system that is the largest provider of online canteen purchasing systems in Australia [35]. A recent review suggests that online ordering platforms can be effective at reducing the energy content of online food purchases [37]. These platforms offer a mechanism to access and provide information to consumers to influence their purchasing decisions. Further research is needed to test different strategies with the potential to improve the purchasing behavior of parents and children who use online canteens.

## 5. Conclusions

In this study involving primary school online canteens, tailored feedback and graphs alone did not increase the proportion of healthy items purchased as a part of lunch orders in a primary school canteen. There was also no improvement in the nutritional quality of lunch orders. Given the potential of online canteen ordering systems to deliver nutrition interventions at scale, further research is recommended into the most effective ways to deliver tailored nutritional information at the point-of-purchase.

## Figures and Tables

**Table 1:** Characteristics of the sample.

	Intervention *n* = 5 Schools	Control *n* = 5 Schools
SCHOOL CHARACTERISTICS ^1^		
Mean (SD) number of enrolments	574.4 (119.3)	460.4 (160.4)
Mean % of Aboriginal or Torres Strait Islander students	6%	12.6%
Socioeconomic status ^2^ *n* (%)		
Least advantaged	3 (60%)	3 (60%)
Most advantaged	2 (40%)	2 (40%)
CANTEEN CHARACTERISTICS ^3^		
Type of canteen operation *n* (%)		
Principal/school run	0 (0%)	4 (80%)
P&F/P&C run	5 (100%)	1 (20%)
Days of operation *n* (%)		
5 days a week	4 (80%)	2 (40%)
3–4 days a week	1 (20%)	3 (60%)
Mean (SD) number of online lunch orders per student per week	0.88 (0.72)	0.84 (0.67)
USER CHARACTERISTICS	*n* = 1499 participants	*n* = 701 participants
Grade of student *n* (%)		
Grade K-2	665 (44.4%)	351 (50.1%)
Grade 3–6	834 (55.6%)	350 (49.9%)
Frequency of use ^4^		
High users (1 or more orders/week) ^5^	609 (40.6%)	258 (36.8%)
Low users (<1 orders/week)	890 (59.4%)	443 (63.2%)

^1^ Based on publicly available school statistics (MySchool 2018); ^2^ SEIFA 2016 data, based on postcode of school locality; ^3^ Based on Canteen Manager survey conducted after the collection of follow-up data; completed by all 10 Canteen Managers; ^4^ Frequency of use based on baseline purchasing characteristics; ^5^ Approximately 1 order/week–4 weeks were included in both the baseline and follow up period.

**Table 2:** Impact of intervention on primary and secondary outcomes in intervention and control groups at follow up, controlling for baseline values.

Variable	Baseline		Follow Up		Intervention vs. Control at Follow-Up	
	Intervention (*n* = 9322 Items)	Control (*n* = 4621 Items)	Intervention (*n* = 8224 Items)	Control (*n* = 4235 Items)	Main Analysis	
					Difference OR/95% CIs	*p*-Value
PRIMARY OUTCOMES						
	% (*n*)	% (*n*)	% (*n*)	% (*n*)		
% of student lunch order items that are ‘everyday’	56.03% (*n* = 5920)	55.90% (*n* = 2795)	58.09% (*n* = 5423)	56.51% (*n* = 2561)	0.99 (0.87, 1.13)	0.88
% of student lunch order items that are ‘caution’	8.81% (*n* = 931)	1.42% (*n* = 71)	8.73% (*n* = 815)	1.24% (*n* = 56)	1.17 (0.73, 1.88)	0.45
SECONDARY OUTCOMES						
	*n* = 5255 orders Mean (SD)	*n* = 2349 orders Mean (SD)	*n* = 4698 orders Mean (SD)	*n* = 2190 orders Mean (SD)		
Energy (kilojoules)	1606 (618)	1837 (729)	1587 (600)	1762 (699)	51 (11, 90)	0.02
Saturated fat (grams)	4 (3)	5 (3)	4 (3)	5 (3)	0.1 (−0.1, 0.3)	0.22
Sugar (grams)	17 (14)	23 (16)	17 (14)	22 (16)	0.1 (−0.7, 1.0)	0.75
Sodium (milligrams)	564 (349)	608 (284)	558 (347)	594 (284)	12 (−9, 33)	0.22
ADVERSE OUTCOME						
Average weekly revenue per school (AUD)	AUD 1477.98 (540.58)	AUD 674.79 (384.38)	AUD 1458.20 (557.20)	AUD 636.86 (399.53)	AUD 18.15 (−89.11, 125.41)	0.74

## Data Availability

The datasets generated during and/or analyzed during the current study are available from the corresponding author on reasonable request, pending ethics approval.

## References

[1] GBD 2019 Risk Factors Collaborators (2020). Global burden of 87 risk factors in 204 countries and territories, 1990–2019: A systematic analysis for the Global Burden of Disease Study 2019. Lancet.

[2] Centers for Disease Control and Prevention Childhood Nutrition Facts. https://www.cdc.gov/healthyschools/nutrition/facts.htm.

[3] Kaikkonen J., Mikkila V., Magnussen C., Juonala M., Viikari J., Raitakari O. (2013). Does childhood nutrition influence adult cardiovascular disease risk? Insights from the Young Finns Study. Ann. Med..

[4] Public Health England (2016). NDNS Results from Years 5 and 6 Combined of the Rolling Programme for 2012 and 2013 to 2013 and 2014.

[5] Australian Bureau of Statistics (2016). Australian Health Survey: Consumption of Food Groups from the Australian Dietary Guidelines.

[6] Australian Institute of Health and Welfare (2018). Nutrition across the Life Stages.

[7] Reedy J., Krebs-Smith S. (2010). Dietary sources of energy, solid fats, and added sugars among children and adolescents in the United States. J. Am. Diet Assoc..

[8] Kreuter M.W., Skinner C.S. (2000). Tailoring: What’s in a name?. Health Educ. Res.

[9] Rimer B., Kreuter M. (2006). Advancing tailored health communication: A persuasion and message effects perspective. J. Commun..

[10] Ulph F., Townsend E., Glazebrook C. (2009). How should risk be communicated to children: A cross-sectional study comparing different formats of probability information. BMC Med. Inf. Decis. Mak..

[11] Ducrot P., Julia C., Mejean C., Kesse-Guyot E., Touvier M., Fezeu L.K., Hercberg S., Peneau S. (2016). Impact of different front-of-pack nutrition labels on consumer purchasing intentions: A randomized controlled trial. Am. J. Prev. Med..

[12] Pettigrew S., Talati Z., Miller C., Dixon H., Kelly B., Ball K. (2017). The types and aspects of front-of-pack food labelling schemes preferred by adults and children. Appetite.

[13] Talati Z., Pettigrew S., Kelly B., Ball K., Dixon H., Shilton T. (2016). Consumers’ responses to front-of-pack labels that vary by interpretive content. Appetite.

[14] Whatnall M.C., Patterson A.J., Ashton L.M., Hutchesson M.J. (2018). Effectiveness of brief nutrition interventions on dietary behaviours in adults: A systematic review. Appetite.

[15] Eyles H.C., Ni Mhurchu C. (2009). Does tailoring make a difference? A systematic review of the long-term effectiveness of tailored nutrition education for adults. Nutr. Rev..

[16] Hamel L.M., Robbins L.B. (2013). Computer- and web-based interventions to promote healthy eating among children and adolescents: A systematic review. J. Adv. Nurs..

[17] Murimi M.W., Nguyen B., Moyeda-Carabaza A.F., Lee H.-J., Park O.-H. (2019). Factors that contribute to effective online nutrition education interventions: A systematic review. Nutr. Rev..

[18] Rocha N., Kim H. (2019). EHealth intervention for fruit and vegetable intake: A meta-analysis of effectiveness. Health Educ. Behav..

[19] Online Food Delivery—Worldwide Statista Market Forecast. https://www.statista.com/outlook/374/100/online-food-delivery/worldwide.

[20] Fiedler J., Eckert T., Wunsch K., Woll A. (2020). Key facets to build up eHealth and mHealth interventions to enhance physical activity, sedentary behavior and nutrition in healthy subjects—An umbrella review. BMC Public Health.

[21] Bates S., Reeve B., Trevena H. (2020). A narrative review of online food delivery in Australia: Challenges and opportunities for public health nutrition policy. Public Health Nutr..

[22] Miller G.F., Gupta S., Kropp J.D., Grogan K.A., Mathews A. (2016). The effects of pre-ordering and behavioral nudges on national school lunch program participants’ food item selection. J. Econ. Psychol..

[23] Delaney T., Wolfenden L., Wyse R. (2021). Online food delivery systems and their potential to improve public health nutrition: response to “A narrative review of online food delivery in Australia”—Letter to the Editor. Public Health Nutr..

[24] Delaney T., Wolfenden L., Yoong S., Sutherland R., Wiggers J., Rissel C., Wyse R. (2018). A cluster randomized controlled trial of a consumer behavior intervention to improve healthy food purchases from online canteens. Asia-Pac. J. Clin. Oncol..

[25] Flexischools (2021). Personal comunication.

[26] Wyse R., Delaney T., Stacey F., Zoetemeyer R., Lecathelinais C., Lamont H., Ball K., Campbell K., Rissel C., Attia J. (2021). The effectiveness of a multi-strategy behavioral intervention to increase the nutritional quality of primary school students’ online canteen lunch orders: The “Click & Crunch” cluster randomized controlled trial. J. Med. Internet Res..

[27] NSW Ministry of Health (2017). The NSW Healthy School Canteen Strategy: Food and Drink Criteria.

[28] National Health and Medical Research Council Australian Guide to Healthy Eating. https://www.eatforhealth.gov.au/guidelines/australian-guide-healthy-eating.

[29] Brug J., Campbell M., van Assema P. (1999). The application and impact of computer-generated personalized nutrition education: A review of the literature. Patient Educ. Couns..

[30] (2016). Commonwealth of Australia; Australian Bureau of Statistics Socio-Economic Indexes for Areas (SEIFA). https://www.abs.gov.au/ausstats/abs@.nsf/mf/2033.0.55.001.

[31] NSW Government FoodFinder. https://www.foodfinder.health.nsw.gov.au.

[32] The George Institute for Global Health FoodSwitch. https://www.foodswitch.com.au/.

[33] Xyris Software Food Works. https://xyris.com.au/products/foodworks-9-professional/.

[34] Australian Curriculum, Assessment and Reporting Authority My School. https://www.myschool.edu.au/.

[35] Delaney T., Wyse R., Yoong S.L., Sutherland R., Wiggers J., Ball K., Campbell K., Rissel C., Lecathelinais C., Wolfenden L. (2017). Cluster randomized controlled trial of a consumer behavior intervention to improve healthy food purchases from online canteens. Am. J. Clin. Nutr..

[36] Niven P., Morley B., Dixon H., Martin J., Jones A., Petersen K., Wakefield M. (2019). Effects of health star labelling on the healthiness of adults’ fast food meal selections: An experimental study. Appetite.

[37] Wyse R., Jackson J.K., Delaney T., Grady A., Stacey F., Wolfenden L., Barnes C., McLaughlin M., Yoong S.L. (2021). The effectiveness of interventions delivered using digital food environments to encourage healthy food choices: A systematic review and meta-analysis. Nutrients.

